# Influenza vaccination coverage of health care workers: a cross-sectional study based on data from a Swiss gynaecological hospital

**DOI:** 10.3205/id000037

**Published:** 2018-02-23

**Authors:** Evelyn Dass von Perbandt, René Hornung, Mirjam Thanner

**Affiliations:** 1 Frauenklinik, Kantonsspital St. Gallen, Switzerland

**Keywords:** pregnancy/gravidity, infectious disease, public health

## Abstract

**Background: **Pregnancy is a risk factor for severe influenza and related complications. The vaccination has been recommended in healthcare workers as a strategy for preventing influenza in risk patients. The aim of this study was to analyze the influenza vaccination rate of the department of obstetrics and gynaecology of the Cantonal hospital St. Gallen in Switzerland.

**Methods:** A cross-sectional study was carried out to investigate the influenza vaccination rates of all staff members of the Department of obstetrics and gynaecology (n=259). The vaccination coverage was compared according to sociodemographic variables using Chi-squared test. Associations were determined using a logistic regression model. Possible reasons for and against vaccination coverage were then investigated.

**Results: **200 questionnaires were included (valid response rate 77%). 15% reported being vaccinated against influenza (n=29). Reasons to be vaccinated are the belief of protection of patients (82%), oneself (75%) or family (61%). Reasons not to get vaccinated, including beliefs regarding the vaccine is not important (49%) and its ineffectiveness (44%). In the logistic regression analysis, the vaccination coverage among doctors (61% vaccinated) and nurses/midwives (4% vaccinated) is different from the vaccination coverage among the non-medical staff reference category (16% vaccinated; p=0.004, p=0.027), after controlling for the effect of other variables sex (p=0.807), age (p=0.438) and full time employment (p=0.298).

**Discussion: **This study showed that doctors have a higher vaccination rate compared to other job roles, whereas the nurses and midwives had very low vaccination rates, which indicate a significant public health communication gap that needs to be addressed.

## Introduction

### Background

Influenza infection is caused by influenza virus, which is known to cause an epidemic annually [1]. Transmitted mainly via droplets, influenza primarily manifests as high fever, malaise, myalgias and arthralgias [1]. In severe cases, pneumonia, acute respiratory disease syndrome (ARDS), secondary bacterial infection and neurologic complications such as encephalopathy and encephalitis can occur [1]. Influenza vaccination is particularly important for people who are at high risk for developing serious influenza-related complications [2]. These include children younger than 5, particularly those younger than 2 years old, pregnant women and elderly (≥65 years of age) and those with chronic medical conditions [2]. 

Annual influenza vaccination is recommended due to antigenic change in circulating influenza virus strains and the relatively short-lived immunity achieved by immunizations [3]. Influenza vaccination has been shown to be moderately protective against influenza [3], with efficacy of 70–90% and has therefore been recommended in healthcare workers (HCWs) [2]. Previous studies have defined HCWs as clinicians, midwives, community-based practice and hospital-based nurses, paramedics, occupational therapists, physiotherapists, radiographers, care-based social workers, community and hospital-based pharmacists, students, trainees and administrative staff based in clinical settings [2]. They (HCWs) comprise a recommended target group for vaccination against seasonal influenza [2]. This recommendation is based on the increased exposure of HCWs to the viruses of seasonal influenza by virtue of their working environment, and hence the increased risk of HCWs transmitting the infection, which could be particularly relevant for patients at risk of serious consequences of infection [2]. 

Some of the typically described adverse events after the influenza vaccination are generalized weakness, nausea, headache, fever and local reactions [4]. But as described by Phengxay et al. in placebo-controlled trials among adults vaccine and placebo groups typically had similar rates of headache, myalgia and malaise [4].

During hospitalization, patients have 5–35 times greater risk of acquiring influenza or an influenza-like illness if exposed to infected patients or HCWs [2]. When assessed within long-term care settings, the benefits of vaccination against influenza, to HCWs have been estimated to be a reduction in days of sick leave of 53% [2].

In the USA, influenza vaccination coverage among residents ranges from 0% to 100%, and averages 80% or higher [5]. Coverage in Europe among residents has been reported to vary from 50–90% [5]. The vaccination rate of HCWs against seasonal influenza in the European Union (EU) varies from 6–54% and vaccination recommendations vary between countries in terms of strength and specificity [2]. In France, Spain, Germany and Italy, the results from a survey carried out in 2011 using qualitative data from face-to-face interviews showed a widespread lack of awareness and understanding among HCWs regarding the importance of vaccination against influenza, which may explain the low level of vaccine uptake [2]. Coverage in the United Kingdom had been described as one of the highest in the EU [2]. Recent data in England showed vaccine uptake by frontline HCWs of 46% and 55% in the 2012/2013 and 2013/2014 influenza seasons respectively [2]. 

### Objectives

The aims of this study were to investigate the influenza vaccination rate of a gynaecological hospital in Switzerland, possible reasons for and against the influenza vaccination and describe possible strategies to promote the influenza vaccination in a non-mandatory setting. 

### Setting 

The department of obstetrics and gynaecology of the Cantonal Hospital, St. Gallen consists of obstetrics (1600 new born babies born in the year 2015), neonatology, gynaecology, gynaecologic oncology, uro-gynaecology and reproductive medicine and employs approximately 260 staff members. Between October and February of every year, the main hospital’s staff doctors’ unit provides free influenza vaccination to all hospital staff members. 

## Methods

In July and August 2015 a cross-sectional study was carried out to investigate the influenza vaccination rates of all staff members of the department of obstetrics and gynaecology and possible reasons for and against vaccination coverage according to the Strengthening the Reporting of Observational Studies in Epidemiology (STROBE) statement (guidelines for reporting observational studies) [6]. A questionnaire was developed using the questionnaire in the study by Heinrich-Morrison et al. [3] (see Attachment 1). A pilot test was conducted among medical staff in the study settings to validate understanding of the questionnaire and its length, as carried out by Godoy et al. [7]. The final questionnaire was carried out in German and contained 10 questions (7 closed and three with closed and open-end answers).

According to the duty roster of July 2015 all staff members of the department of obstetrics and gynecology, who were planned on duty, received a personally addressed envelope containing the questionnaire in their personally designated post box. The questionnaires were distributed on the 1^st^ of July and the deadline for all responses was set on the 31^st^ of July 2015. Approval to perform this study was obtained from the head of department. Participation was voluntary and anonymity of the respondents was preserved. There were no eligibility criteria set.

After data collection, the rates of influenza vaccination were compared according to sex, age, job role, employment, and direct contact with patients using the Chi-squared test, with p<0.05 deemed statistically significant. The vaccination coverage was determined using a logistic regression model with input of sociodemographic variables with a significance of p<0.05. The analysis was performed using the SPSS Version 20.

## Results

228 questionnaires were received from a total of 259 employees planned on duty in July 2015 at the department of obstetrics and gynaecology. This corresponded to a first response rate of 88%. One questionnaire was excluded, as the vaccination status of the respondent was not stated. The total number of 27 questionnaires of apprentices, interns and students were excluded due to group heterogeneity and fluctuation. Eight questionnaires were not completed fully: Information on the level of employment (n=1), direct contact with patients (n=2) and age (n=5) was missing. Nevertheless, these questionnaires were included in the analysis. The total valid response rate was 77%.

Of the 200 total valid respondents 94% were female (n=188). As reported by the study of Heinrich-Morrison et al. [3], majority of respondents in this study were nurses or midwives (n=137, 69%) followed by non-medical staff members (n=32, 16%) and doctors (n=31, 16%) and more than half of the staff (55%, n=109) worked on a part time basis. The average age of all respondents was 39 years (median 37 years, minimum 20 years and maximum 62 years). 91% (n=180) reported regular direct contact with the patients. 

**Figure 1:**
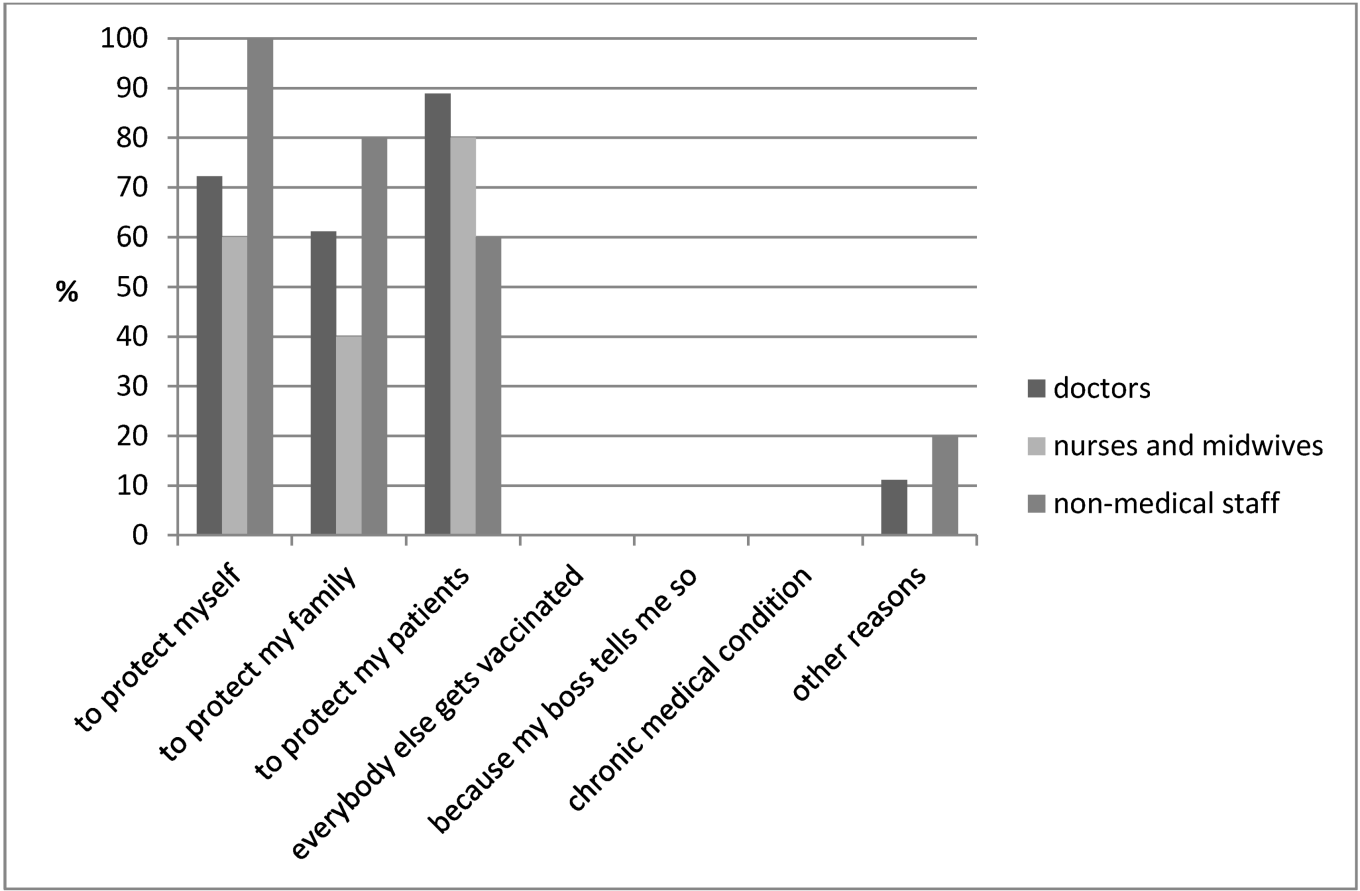
Vaccinated healthcare workers: reported reasons for vaccination (28 staff members responded to the question)

75.8% of the respondents in the study of Heinrich-Morrison et al. [3] self-reported being vaccinated against influenza. In the present study merely 15% of respondents (n=29) were vaccinated against influenza. Figure 1 (Fig. 1) illustrates reasons opting for vaccination by healthcare workers, including to protect their patients (doctors 89%, nurses and midwives 80%) and self-protection (doctors 72%, nurses and midwives 60% and non-medical staff 100%). 28 staff members responded to the question. 

62% of the vaccinated (n=18) reported of being vaccinated at the main hospital’s staff doctors’ unit, 10% (n=3) reported being vaccinated at the general practitioner or other doctor, 17% (n=5) reported to vaccinate themselves and 10% (n=3) reported to be vaccinated in other institutions. Table 1 (Tab. 1) summarizes the characteristics of the study participants and their vaccination coverage.

In the logistic regression analysis, the vaccination coverage among doctors (p=0.004) and nurses/midwives (p=0.027) is different from the vaccination coverage among the non-medical staff reference category, after controlling for the effect of other variables sex (p=0.807), age (p=0.438), and full time employment (p=0.298). Table 2 (Tab. 2) summarizes the logistic regression analysis.

**Figure 2:**
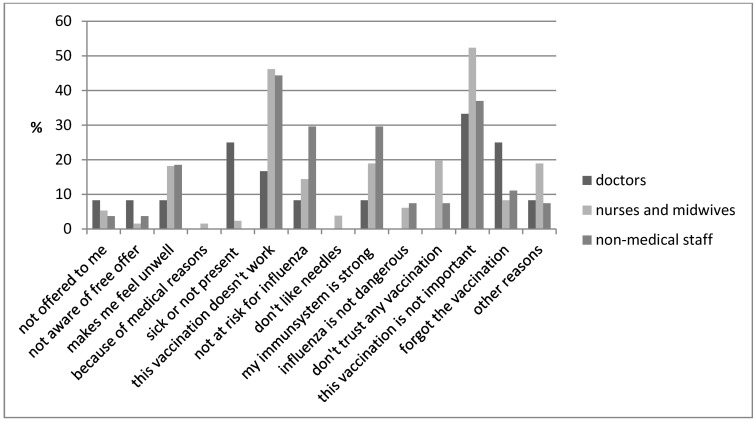
Not vaccinated healthcare workers: reported reasons for choosing not to be vaccinated (171 staff members responded to the question)

Figure 2 (Fig. 2) summarizes the reasons for staff members not to get vaccinated according to job roles, including beliefs regarding the vaccine is not important (doctors 33%, nurses and midwives 52% and non-medical staff 37%) and its ineffectiveness (doctors 17%, nurses and midwives 46% and non-medical staff 44%). 

**Figure 3:**
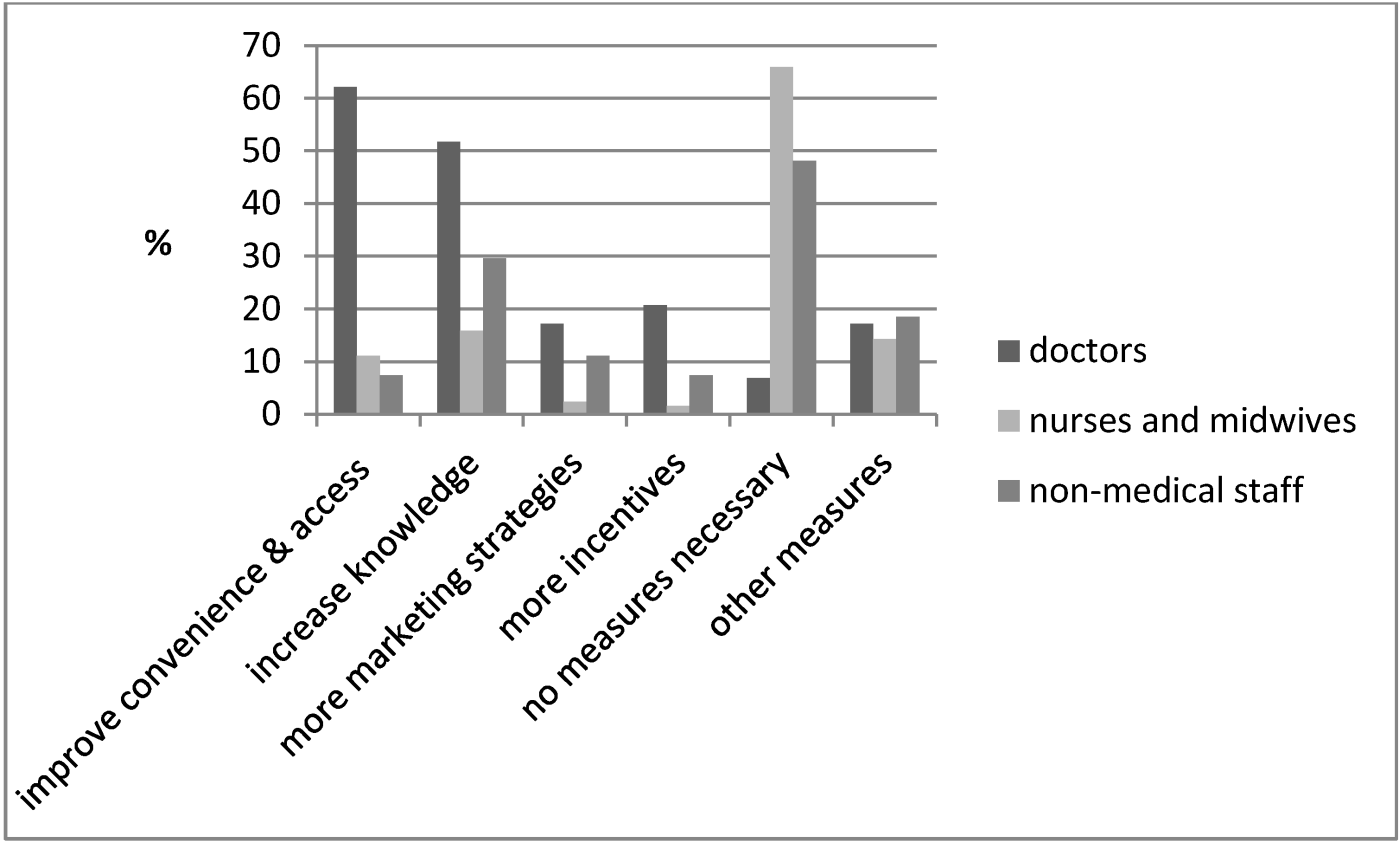
Strategies reported that could improve vaccination coverage (182 staff members responded to the question)

One of the objectives of this study was to describe possible strategies to promote the influenza vaccination in a non-mandatory setting. The last question of the questionnaire asked respondents of their opinion, what strategies they thought could improve the influenza vaccination rate. The strategies stated in the questionnaire described components of a staff influenza vaccination program carried out in the study of Heinrich-Morrison et al. [3]. The components included vaccine availability, communication, marketing and incentives. Figure 3 (Fig. 3) summarizes the opinions of the respondents, including improving convenience and access and increasing knowledge (62% and 52% of doctors’ opinions), whereas no measure necessary by 66% of nurses and midwives’ opinions.

## Discussion

Pregnant women are a high risk for influenza virus infection and serious influenza-related complications and are recommended to receive inactivated influenza vaccine during the influenza season [1]. Healthcare workers may be exposed to the influenza virus in the workplace and can also act as a source of infection of patients [7]. Evidence suggests that seasonal influenza vaccination protects the healthcare workers and improves outcomes in patients, including evidence of mortality reduction [8]. 

Despite explicit recommendations by public health authorities and studies stating that annual influenza vaccination of healthcare workers is associated with a reduction of morbidity and mortality among patients [8], [9], vaccination rates among HCWs worldwide are low, with only about 4–40% coverage rates being achieved [9]. In 2014 a survey was carried out through a web-based platform with protected access restricted to nominated experts from each EU/EEA Member States [10]. The survey was a collaborative study between the European Centre for Disease Prevention and Control (ECDC), the Vaccine European New Integrated Collaboration Effort (VENICE) Project and the EU/EEA Member States [10]. Influenza vaccination rates for HCWs for the 2011–12 and 2012–13 seasons were provided by 13 Member States [10]. The median vaccination rate in 2012–13 was 28.6% [10]. The highest vaccination rates were reported by the United Kingdom – England (45.6%), Romania (42%), Lithuania (36.6%), United Kingdom – Wales (35.5%) and United Kingdom – Scotland (33.7%) [10]. Although the vaccination rate for the Netherlands was also high (75%), it was only calculated for general practices and is overestimated [10]. Ireland and Portugal reported vaccination rates among HCWs working in long-term healthcare settings with 15% and 27%, respectively [10]. Vaccination rates in Switzerland were relatively low, with studies finding about 15% coverage rates among nurses [9]. 

This is one of the few studies to investigate influenza vaccination rates and seek views of HCWs (doctors, nurses/midwives) on their own uptake of seasonal influenza vaccination in a gynaecological department with high-risk patients (pregnant women and infants). 15% of respondents (n=29) in the present study were reported being vaccinated against influenza, similar to the rates described in Switzerland earlier [9]. Key reasons for getting vaccinated in the present study were to protect themselves, their patients and their own family. These reasons were also stated in the study by Ishola et al. [8]. Shrikrishna et al. went on to show a significant difference in vaccination rates between staff groups, with doctors being the most likely to have been vaccinated [11]. The regression model in the present study also reported that doctors had a higher vaccination rate compared to other job roles, and nurses and midwives had very low vaccination rates. This could in turn suggest some variation in attitudes between staff groups, which may be relevant for developing approaches to improve uptake [11].

In review by Hollmeyer et al., more than 90% of studies reviewed from 1980 to 2008 showed that HCWs stated self-protection being the most important reason for vaccination acceptance [12]. In the present study, the reasons for vaccination coverage focused more on protection of patients than self-protection. Hollmeyer et al. identified further two major reasons for lack of vaccine uptake by HCWs. Firstly a wide range of misconceptions or lack of knowledge about influenza infection and secondly, a lack of convenient access to vaccine [12], comparable to the typical reasons of vaccinated staff members in the present study. Some of the not-vaccinated members of staff in this study also felt that no measures were necessary. Improving staff education, especially based on changing negative attitudes of HCWs, the perception that the influenza immunization does not work, that staff may not be at risk of influenza and adverse effects of the immunization should be urgently addressed and may increase vaccination coverage [13], [14]. 

Furthermore providing evidence-based arguments about the safety of new vaccines and the priority of public health over personal choice, and creating strong social norms for influenza vaccination as part of the organizational culture, could increase uptake of influenza vaccination among primary care HCWs and their patients [15].

Several different strategies have been suggested by literature. A study carried out by Dorribo et al. investigated the mask-wearing policy, which was a motivation for vaccination but also offered an alternative to non-compliant HCW [16]. As described in their study, concerns about vaccine safety and efficiency and self-interest of HCWs were main determinants for influenza vaccination acceptance [16]. Better incentives were recommended to encourage vaccination amongst non-physician HCW [16]. Shrikrishna et al identified potential barriers to vaccination and suggested that action should focus in particular on increasing the convenience of vaccination for staff [11].

In the study by Pless et al. [9], three interconnected themes were identified, why nurses in that study declined influenza vaccination. Firstly, the idea of maintaining a strong and healthy body, which was a central motif for rejecting the vaccine [9]. Secondly, the wish to maintain decisional autonomy-especially over one’s body and health [9]. Thirdly, nurses' perception of being surrounded by an untrustworthy environment, which restricts their autonomy and seemingly is in opposition to their goal of maintaining a strong and healthy body [9]. According to their study, nurses tend to rely on conventional health beliefs rather than evidence based medicine when making decision to decline influenza vaccination [9]. It seemed important to identify and acknowledge these interrelations. In order to reach nurses as in their study, they suggested interventions to increase influenza vaccination should be tailored specifically for a certain group instead of applying a “one size fits all” approach [9]. The teaching of evidence based decision-making should be integrated on different levels, including nurses’ training curricula, their workspace and further education [9]. Pless et al. described in a further article, that filling in of declination forms or mandatory influenza vaccinations as a condition of employment seemed to be the most accepted enforced measures [17]. As declination forms have been shown to be of less effect on overall patient protection, they advocated mandatory influenza vaccination as a condition of new (and perhaps ongoing) employment as a feasible, effective and ethical measure to increase vaccination rates [17]. 

In the USA, the Center for Disease Control and Prevention influenza season report from 2014/15 showed that 77,3% of all HCWs reported having had an influenza vaccination, an increase of 13,8% compared with the 2010/11 season estimate [18]. This improvement in vaccine coverage was attributed mainly to more hospitals adopting mandatory vaccination policies [18].

Heinrich-Morrison et al. described other data from US centres that suggested promotion of vaccination in settings where vaccination is not required (i.e. non-mandatory), could significantly increase uptake and especially when these effectively engage medical staff [3]. Heinrich-Morrison et al. carried out a survey containing 10 question regarding the influenza vaccination status and barriers and enablers to influenza vaccination [3]. HCWs of their study, who opted to remain unvaccinated, cited comparable reasons to the present study, including beliefs regarding vaccine ineffectiveness, that vaccination makes staff unwell, and that vaccination being not required because staff was at low risk for acquiring influenza [3]. Based on the information collected, a HCW influenza vaccination program was implemented the following year, consisting of components, including vaccine availability, communication, marketing, database and reporting and incentives [3]. There was a significant improvement shown in vaccination against influenza overall and amongst all staff categories with clinical contact [3]. 

Non-vaccination interventions, including antiviral prophylaxis and treatment and social distancing measures such as school closure, could complement the influenza vaccination, improving the effectiveness and cost effectiveness of both pre-emptive and reactive vaccination [19]. As concerns about the efficacy of the influenza vaccine remain and healthcare costs continue to rise, over the counter medicines i.e. non-prescription medicines may play an increasingly important role in mitigating the socioeconomic burden of this pervasive seasonal illness [20]. For individuals with mild to moderate influenza symptoms, over the counter medicines allow early cost-efficient self-treatment and patient control [20]. They also offer convenience, wide availability, and a range of treatment choices [20]. Socioeconomically, over the counter medicines can reduce use of the healthcare system and contribute to increased economic productivity by reducing time absent from work [20].

Contrary to the study carried out by Haridi et al., where 86.9% of the physicians and 93.3% of the nurses were described vaccinated [18], the results of the present study showed a significant difference in the vaccination rates between the different HCW groups. Of the 15% of the self-reported vaccinated HCWs in this study, 61% were doctors and 4% nurses/midwives. The majority of the doctors in this study were of the opinion that improving the convenience and accessibility of the influenza vaccination for example on wards or at multi-discipline meetings (62%), increasing knowledge about influenza and preventing it through vaccination (52%) and more incentives such as free coffee during vaccination (21%) could improve the vaccination rates among HCWs. Only a small percentage of the nurses and midwives were of this opinion (improving convenience and accessibility 11% and increasing knowledge 16% could increase influenza vaccination rates). Despite the low vaccination rates of nurses and midwives (4%), more that 66% of this group of HCWs were of the opinion, that no measures were necessary. These results imply that over half of the nurses/midwives were of the opinion, that none of the strategies suggested could increase the influenza vaccination rates in this study. The importance of identifying and acknowledging interrelations in the different HCWs and tailoring interventions for each HCW group instead of applying a “one size fits all” approach as mentioned earlier, could be the key to increasing the vaccination rates [9].

### Limitations

The present study is a cross-sectional study that describes vaccination rates of influenza in a gynaecological department and seeks views of HCWs (doctors, nurses/midwives and administrative staff members) on their own uptake of seasonal influenza vaccination. Cross-sectional studies are a common research method to acquire information; a valuable snapshot of the respondents’ views [8]. However the limits of any cross-sectional studies apply to this study; for example, it is unable to assess possible changes and shifts in views and opinions after strategies over time [8].

Further limitations such as sources of potential bias could be discussed in the selection of participants and the vaccination rate of the respondents of this study. Like other interview studies, and described in studies such as Ischola et al. [8] and Pless et al. [17], the present study relied on consenting participants, increasing the chance of a biased sample; staff members who came forward may have been more likely to be unvaccinated healthcare workers with a more pronounced opinion on this topic and who are particularly interested in immunization issues [8], [17]. It remains difficult to state how representative the sample in the present study was. 

Nevertheless the findings of this study provide a useful insight into the views of influenza vaccination for future strategy and intervention-makers. The study design and the modified questionnaire developed in this study in accordance to the set-up of this study could be useful for extended surveys of vaccination rates in further departments of the Cantonal Hospital of St. Gallen with high risk patients such as haematology, cardiology, nephrology, geriatrics, ICU, oncology as described in the study by Pless et al. [9] or other gynaecological departments of other hospitals in Switzerland or in Europe. The information attained in this study could serve as a baseline from which further exploration in this subject can take place in the future.

## Conclusion

Pregnant women are at high risk for influenza virus infection and serious influenza-related complications and are recommended to receive inactivated influenza vaccine during the influenza season [1]. Maternal influenza immunization is promising strategy to reduce morbidity and mortality associated with influenza among pregnant women and young infants [21]. 

The unpredictability of influenza makes it difficult to forecast severity and timing from year to year [11]. However, a successful influenza vaccination programme could help protect staff and reduce the risk of transmission to patients in their care, who may not have been vaccinated [11].

As described in the study by Ishola et al., much is known about HCW in general, but there is very limited information specifically on midwives, who are probably the most influential group of HCWs with regard to pregnant women [8]. Seasonal influenza vaccine uptake by midwives has been shown to be much lower than among other HCWs [8]. The present study showed comparably low vaccination rates of nurses and midwives, outlining a target group that needs to be addressed. 

Proposals for better encouragement with midwives through direct, open and transparent communication and practical suggestions for working with staff to develop more effective publicity and provision arrangements for work-based seasonal influenza vaccination could contribute to boost vaccine uptake levels [8]. Mandatory influenza vaccination as an obligatory requirement for HCW could be considered, although most midwives in other studies and in the present study disagreed to this intervention [8]. 

In summary, low influenza vaccination rates of HCWs remains a huge problem and indicates a significant public health communication gap. Such studies as the present study, could play an important role in investigating other vaccination rates and describing further strategies to improving the vaccination rates. 

## Notes

### Acknowledgement

We thank all participants and staff members of the department of obstetrics and gynaecology of the Cantonal Hospital of St. Gallen. 

We also thank Dr. Rafael Sauter (Clinical Trials Unit, Kantonsspital St. Gallen) for his support.

### Competing interests

The authors declare that they have no competing interests.

## Supplementary Material

Questionnaire

## Figures and Tables

**Table 1:**
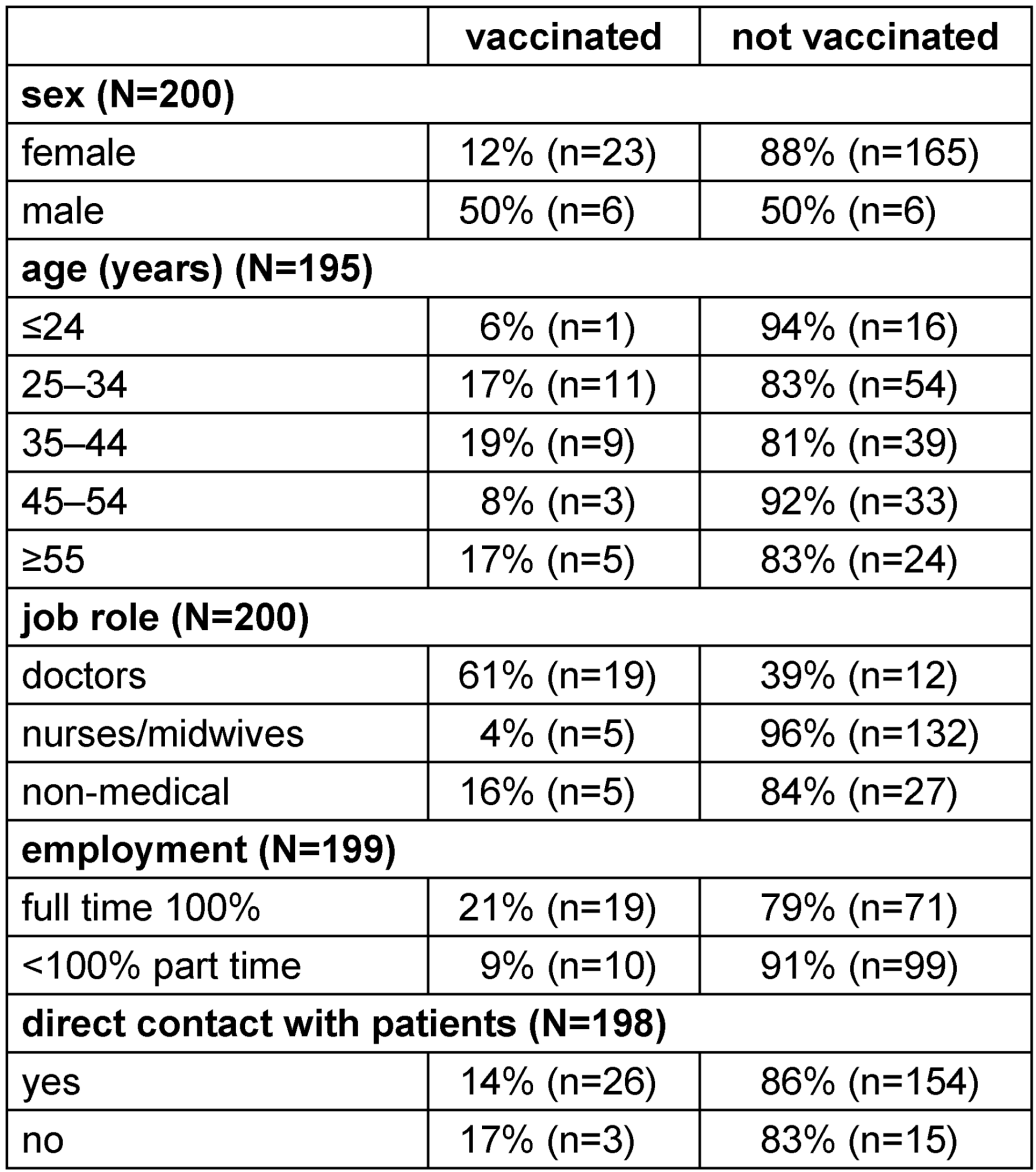
Characteristics of vaccinated and not vaccinated staff members

**Table 2:**
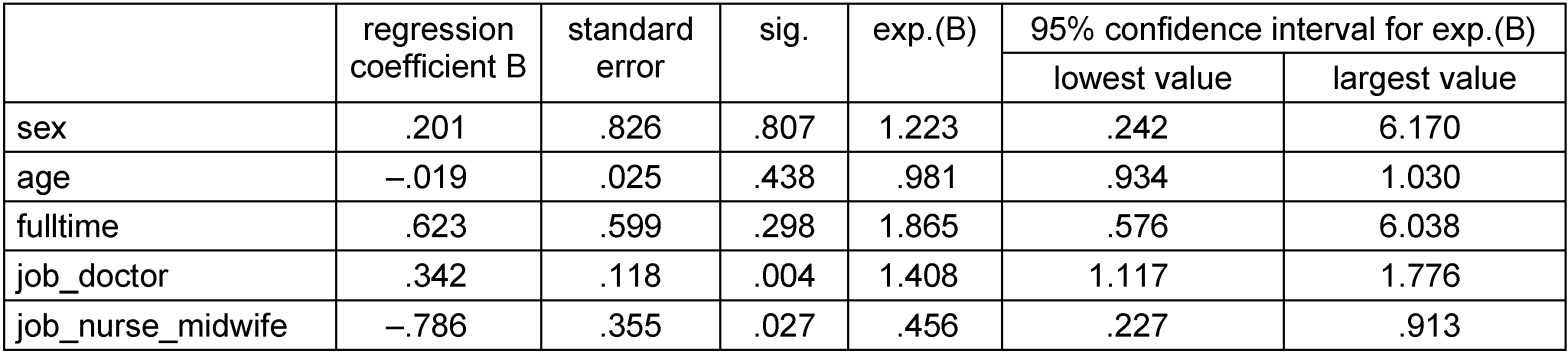
Factors of staff members associated with their influenza vaccination in the logistic regression model
