# Supplementary material for: Influenza vaccination coverage of health care workers: a cross-sectional study based on data from a Swiss gynaecological hospital
Source: GMS Infect Dis. 2018 Feb 23;6:Doc02. doi: 10.3205/id000037 (PMC6301741; doi:10.3205/id000037)
Supplement: Questionnaire [file ID-06-02-s-001.pdf]

Attachment 1 to: Dass von Perbandt E, Hornung R, Thanner M. Influenza vaccination coverage of health care workers: a cross-sectional study based on data from a Swiss gynaecological hospital. GMS Infect Dis. 2018;6:Doc02. DOI: 10.3205/id000037

Questionnaire

1. What is your job role?
doctor
nurse
midwife
medical student, apprentice, practical student
non-medical personnel

2. Do you have direct contact with patients?
yes
no

3. Are you male or female?
male
female

4. In which year were you born?

5. Do you work full time or part time?
full time
part time ≥50%
part time <50%

6. Were you vaccinated against influenza in the season 2014/2015?
yes. Please go to Question 7, 8 and 10
no. Please go to Question 9 and 10

7. Where were you vaccinated?
in the staff immunisation clinic
at a general practice or other doctor
I ordered the vaccination and vaccinated myself
other (please clarify)

8. What are good reasons to be vaccinated?
to protect myself
to protect my family
to protect my patients
because everybody else gets vaccinated
because my boss tells me so
I have a chronic medical condition
other reasons (please clarify)

9. Why didn't you vaccinate yourself against influenza this year?
it was not offered to me
I am not aware of the free offer at the cantonal hospital
it makes me feel unwell
I cannot have it for medical reasons (e.g. allergy)
I was sick or not present on the day it was offered
I do not think this vaccination works
I do not think I am at risk for influenza
I do not like needles
my immune system is strong, I won't get influenza
Influenza is not dangerous
I do not trust any vaccinations
I think vaccinations are important, but I do not think the influenza vaccination is important
I forgot to get vaccinated
Other reasons (please clarify)

10. Which strategies could improve the influenza vaccination rates?
Improve convenience and accessibility e.g. vaccine availability on ward and during routine multidisciplinary meetings
Increase knowledge e.g. notices and short presentations
More marketing strategies e.g. small campaign pins, posters
More incentives e.g. free coffee during immunisation, awards for the most immunised departments/wards
No measures necessary
Others (please clarify)
